# Generation of Type I Collagen Gradient in Polyacrylamide Hydrogels by a Simple Diffusion-Controlled Hydrolysis of Amide Groups

**DOI:** 10.3390/ma3042393

**Published:** 2010-03-26

**Authors:** Masaya Yamamoto, Kaoru Yanase, Yasuhiko Tabata

**Affiliations:** Department of Biomaterials, Institute for Frontier Medical Sciences, Kyoto University 53 Kawara-cho Shogoin, Sakyo-ku, Kyoto 606-8507, Japan; E-Mails: masaya@frontier.kyoto-u.ac.jp (M.Y.); ma-pyu@umin.net (K.Y.)

**Keywords:** gradient, diffusion-controlled hydrolysis, collagen, cell attachment, hydrogel

## Abstract

The objective of this study is to develop an easy and simple diffusion-controlled fabrication technique to generate the concentration gradient of biomolecules in hydrogels. Polyacrylamide (PAAm) hydrogels with a concentration gradient of type I collagen were prepared to evaluate the cell adhesion. The PAAm hydrogel was exposed to a gradient concentration of sodium hydroxide (NaOH) solution at 52 °C to generate that of carboxyl groups in the hydrogel. The carboxyl groups generated were chemically coupled with the amino groups of type I collagen to prepare the hydrogel with a concentration gradient of collagen immobilized. The attachment of L929 fibroblasts was evaluated for the collagen-immobilized hydrogel prepared. The amount gradient of carboxyl groups in the hydrogel increased with an increase in the NaOH concentration while the carboxyl groups gradient enabled to generate a gradient of collagen immobilized in the hydrogel. On the other hand, the number of fibroblasts adhered depended on the amount of collagen immobilized. These findings indicate that the adhesion behavior of cells is modified by the concentration gradient of biomolecule in the three-dimensional scaffold of cells.

## 1. Introduction

Tissue engineering is a technology or methodology to assist the induction of tissue regeneration at defective tissues or organs by either transplanting cells or mobilizing host cells of high potential bioactivities. To successfully implement cell-induced tissue regeneration, however, it is necessary to create a local environment that promotes cell proliferation and differentiation [1]. Several three-dimensional materials have been explored either as potential scaffolds for the cell proliferation and differentiation and/or as delivery vehicles for growth factors that can enhance the cellular activities for tissue regeneration [2].

Many researches have demonstrated that the scaffolds and growth factors effectively function to enhance the regeneration of various tissues [3]. However, new technologies and methodologies are required to realize the regeneration of tissue with more complex architectures, such as gradient tissue structures at the interface between the hard and soft tissues. It is well recognized that most tissue architectures are greatly influenced by native cellular environments which are mainly regulated with the three-dimensional assignment of biomolecules [4]. As the trial to achieve the regeneration of tissues with a histological gradient structure, it is interesting to develop a tissue engineering technique that can create biomolecule gradients in cell scaffolds.

Multicellular processes of development are often sophisticatedly regulated by the spatial arrangement of extracellular matrix (ECM) and signaling molecules in a time- and concentration-dependent manner. Among them, the concentration gradients of biomolecules, such as growth factors and transcriptional factors, play a pivotal role in the *in vivo* induction and formation of tissues and organs with complex structural architectures [4]. Therefore, if a three-dimensional scaffold with a concentration gradient of biomolecules can be designed, it may allow cells to induce the regeneration of tissue with a natural architecture.

Several methods have been investigated to create the two-dimensional gradients of protein [5]. The most well known are soft lithographic techniques, including microcontact printing [6,7], microfluidic patterning [8,9,10], and photomasking [11,12,13], although the fabrication of protein gradients immobilized on two-dimensional surfaces has still been a technical challenge [5]. Moreover, little has been demonstrated to develop a significant technology and methodology to fabricate scaffolds with a three-dimensional concentration gradient, whereas there has been reported on some complex techniques, such as assembling different microspheres containing biomolecules with a gradient mixing ratio [14] and mixing different solutions by using a gradient maker normally used to make polyacrylamide gels for electrophoresis [15,16,17].

In this study, a diffusion-controlled fabrication technique was developed as an easy and simple method to generate the gradients of functional groups in three-dimensional scaffolds. Polyacrylamide (PAAm) hydrogels were employed as a model material for the generation of functional group gradients because the amide group in the PAAm hydrogel can be mildly hydrolyzed into the carboxyl group which is highly susceptible to further chemical conjugation with biomolecules. The concentration gradients of carboxyl groups were generated in the PAAm hydrogel under different hydrolysis conditions, and chemical conjugation of the carboxyl groups with the amine groups of type I collagen was performed to prepare the hydrogel with a concentration gradient of type I collagen. We examined the attachment and morphology of fibroblasts on the collagen-immobilized hydrogels *in vitro*. This study experimentally confirms that the present diffusion-controlled fabrication technique is effective in fabricating scaffolds with a three-dimensional concentration gradient of biomolecules.

## 2. Results and Discussion

### 2.1. Diffusion-controlled fabrication technique

**Figure 1:**
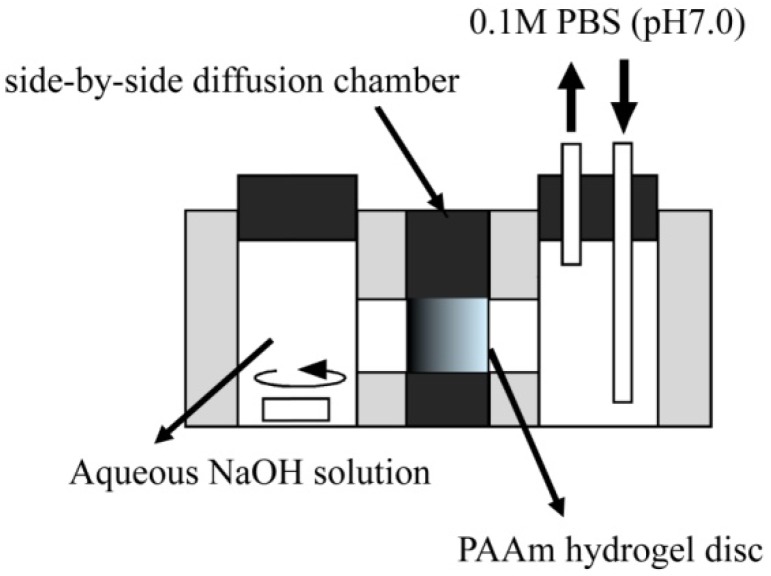
Schematic representation of a side-by-side diffusion chamber to generate a concentration gradient of carboxyl groups in PAAm hydrogels.

**Figure 2:**
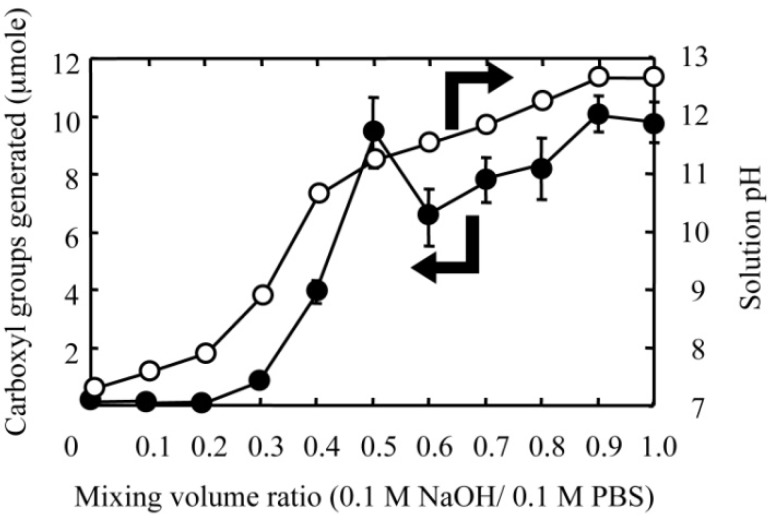
Generation of carboxyl groups in PAAm hydrogels in different concentrations of NaOH solution: (○) the amount of carboxyl groups generated in PAAm hydrogels and (●) the pH value of solutions at different concentrations of NaOH.

The diffusion-controlled fabrication technique could generate the three-dimensional gradients of functional groups in hydrogels. Carboxyl group gradients in PAAm hydrogel discs were generated by a diffusion-controlled hydrolysis of amide groups, which can be realized by the formation of a sodium hydroxide (NaOH) concentration gradient in the hydrogel (Figure 1). In the side-by-side diffusion chamber, both the NaOH and 0.1 M phosphate-buffered solution (PBS, pH 7.0) diffuse across the hydrogel disc in opposite directions and are mixed in different ratios based on their local concentration in the hydrogel. The pH value of NaOH solutions after mixing with PBS decreased with an increase in the mixing volume ratio of PBS to NaOH, while the amount of carboxyl groups generated in the hydrogel disc depended on the pH value of mixtures (Figure 2). This result suggests that the local concentration of NaOH in the hydrogel disc affected the hydrolysis extent of amide groups into carboxyl groups. Therefore, both the initial NaOH concentration and the reaction time for the hydrolysis of amide groups are key to control the concentration gradients of carboxyl groups.

**Figure 3:**
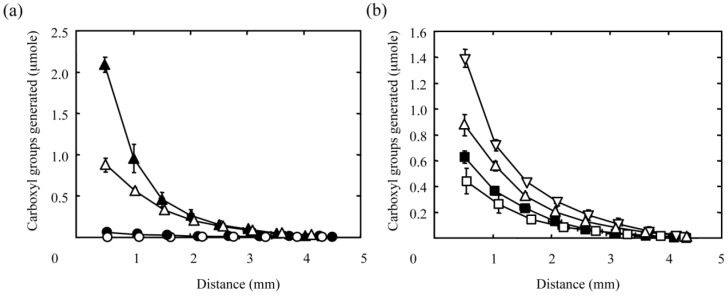
Effect of the NaOH concentration (a) and reaction time (b) on the amount of carboxyl groups generated in PAAm hydrogels by the hydrolysis of amide groups with NaOH solution. (a) The concentration of the NaOH solution is 0.001 (○), 0.01 (●), 0.1 ( △), or 1 M (▲). The reaction time is 30 min. (b) The reaction time is 10 (□), 20 (■), 30(△), or 60 min (▽). The concentration of the NaOH solution is 0.1 M. The X-axis indicates the distance from the surface of hydrogel facing the NaOH solution.

Figure 3 shows the concentration gradients of carboxyl groups generated in PAAm hydrogel discs by the diffusion-controlled hydrolysis of amide groups both with different NaOH concentrations (Figure 3a) and reaction times (Figure 3b). Irrespective of the reaction conditions, the amount of carboxyl groups generated decreased gradually with distance from the surface of hydrogel disc facing the NaOH solution. Every curve in Figure 3a represents a gradient prepared by the hydrolysis reaction for 30 min. It is clear that the increasing concentration of NaOH solution resulted in larger gradients in terms of the amount of carboxyl groups generated in the hydrogel disc. Furthermore, the amount of carboxyl groups generated increased with reaction time (Figure 3b). These results indicate that the diffusion-controlled hydrolysis under the different reaction conditions allows us to alter the concentration gradients of carboxyl groups in the hydrogel disc.

In general, the Fick’s law will be of considerable practical value in predicting the rate of molecular transports in a multi-component system. However, the profiles of carboxyl group gradients are totally different from that of local NaOH concentrations predicted by the Fick’s law. This is probably due to the hydrolysis reaction simultaneously occurring with the diffusion of NaOH in PAAm hydrogels. Other parameters, such as the acrylamide monomer concentration and crosslinking density of PAAm hydrogels, are considered to be important to modulate the diffusion-controlled hydrolysis. The diffusion chamber meets a technical difficulty in treating PAAm hydrogels with lower Young’s moduli prepared at lower monomer concentrations and crosslinking densities, and further technical development should be made.

### 2.2. Fabrication of hydrogels with a concentration gradient of type I collagen

**Figure 4:**
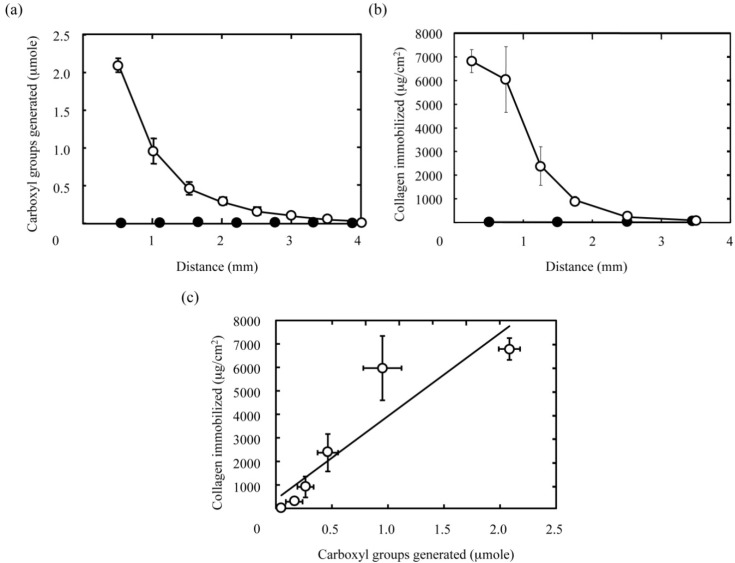
The immobilization pattern of type I collagen in PAAm hydrogels with a concentration gradient of carboxyl groups. (a) The concentration gradient of carboxyl groups in PAAm hydrogels generated by the hydrolysis for 30 min with 1 M NaOH (○) and NaOH-free solutions (●). The X-axis indicates the distance from the surface of hydrogel facing the NaOH solution. (b) The amount of collagen immobilized in the corresponding hydrogels to (a). (c) The relationship between the amounts of carboxyl groups generated and collagen immobilized in PAAm hydrogels. The coefficient of determination, R^2^ value for the least mean square fitting is 0.842.

The carboxyl groups generated in the longitudinal slice of PAAm hydrogel discs were chemically coupled with the amino groups of type I collagen by using 1-ethyl-3-(3-dimethylaminopropyl) carbodiimide hydrochloride (EDC) and N-hydroxysuccinimide (NHS) to prepare the hydrogel slice with a concentration gradient of collagen immobilized. The type I collagen immobilized in the hydrogel slice was stained with Sircol dye reagent for further quantitative analysis. Figure 4 shows the concentration gradient of type I collagen immobilized in the hydrogel slice. As shown in Figure 4b, the amount of type I collagen immobilized gradually decreased with distance from the surface of hydrogel disc facing the NaOH solution, while type I collagen was not detected in hydrogel discs without carboxyl groups. It is well known that the surface of PAAm-grafted materials exhibits poor protein adsorption [18]. As a result, non-specific adsorption of type I collagen to the matrix of hydrogels was not observed as seen in Figure 4b. In other words, we can say that the immobilization reaction is highly limited to the carboxyl groups generated (Figure 4a). This high selectivity in immobilization leads to the well correlation between the amounts of the collagen immobilized and the carboxyl groups generated as seen in Figure 4c. Taken together, these results strongly suggest that protein gradients could be introduced into hydrogels by altering the gradient of functional groups in a well-controlled manner.

### 2.3. Cell responses to hydrogel slices with a concentration gradient of type I collagen

**Figure 5:**
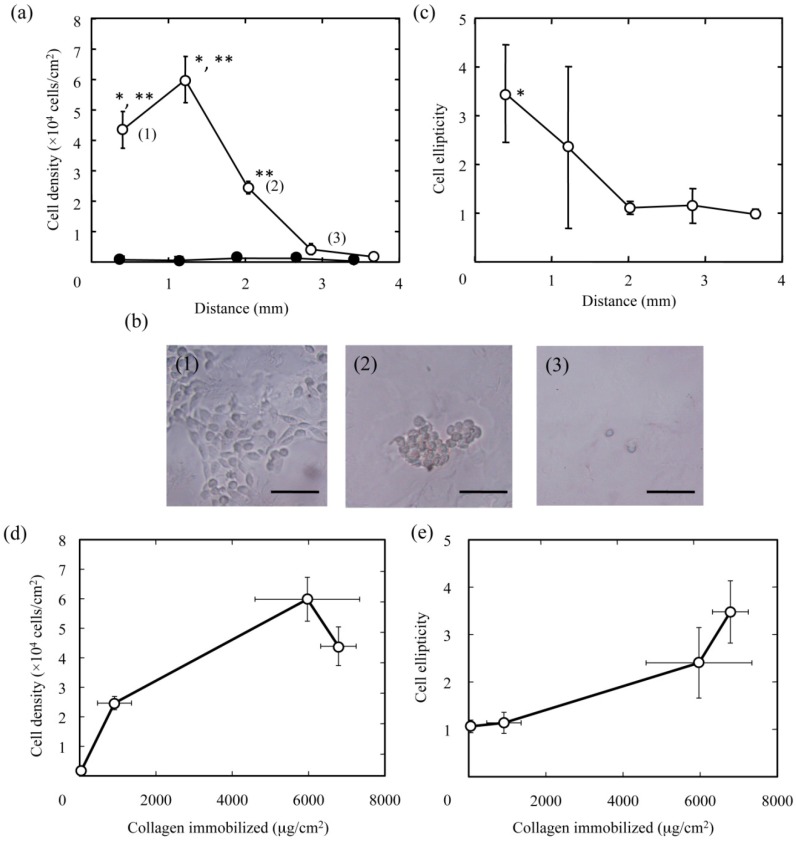
The attachment and morphology of L929 cells on PAAm hydrogels with a concentration gradient of type I collagen. (a) Effect of the concentration gradient of collagen immobilized in the PAAm hydrogel on the cell density. * p < 0.05; significance against the cell density of the plot (2) in the figure. ** p < 0.05; significance against the cell density of the plot (3) in the figure. (b) Phase contrast microscopic pictures of cells attached at the sites which correspond to the plots (1), (2), and (3) in Figure 5a. Bars correspond to 100 μm. (c) Effect of the concentration gradient of collagen immobilized in the PAAm hydrogel on the cell ellipticity calculated by image analyses for the microscopic pictures of cells (Figure 5b). * p < 0.05; significance against the cell ellipticity at the site of 2 mm and longer in distance from the surface of hydrogel facing the NaOH solution. (d) Effect of the amount of type I collagen immobilized in the PAAm hydrogel on the cell density. (e) Effect of the amount of type I collagen immobilized in the PAAm hydrogel on the cell ellipticity.

Cell responses to hydrogel slices with a concentration gradient of type I collagen were investigated with L929 mouse fibroblasts. As shown in Figure 5a, it was found that the cell density significantly decreased with distance from the surface of hydrogel discs facing the NaOH solution. More cells adhered as the type I collagen amount increased (Figure 5d). Similar to the cell density, the morphology of cells adhered varies with the amount of type I collagen immobilized. The morphology of cells adhered became more spreading with a spindle shape (Figure 5b). In addition, the cell ellipticity, which was defined as the major to minor axis ratio, was calculated by measuring the length of those axes for the cells attached (Figure 5c). Significantly higher cell ellipticity was found in the site (1) with the highest amount of type I collagen immobilized (Figure 5e).

Tamada *et al.* have reported that the collagen-immobilized surface facilitates the adhesion, proliferation, and collagen synthesis of fibroblasts *in vitro* within three days [19], suggesting that fibroblasts can secrete their extracellular matrices to create their own microenvironment when they are sitting on a substrate suitable for their phenotype expression. On the basis of this finding, it is conceivable that the initial cell behavior directly influenced by the type I collagen gradients on a substrate could play an important role to determine the following cell fate.

Recently, some researches have demonstrated that two-dimensional concentration gradients of biomolecules affect the alignment and migration of cells *in vitro* [5,6,7,8,9,10,11,12,13,15,16,17,20]. Smith *et al.* showed that the drift speed for bovine aortic endothelial cells is faster on the fibronectin gradient substrate than that on the fibronectin constant substrate [20]. A concentration gradient of Arg-Gly-Asp-Ser (RGDS) peptide on polyethyleneglycol-based hydrogel facilitates fibroblasts alignment along the RGDS-gradient axis and fibroblasts migration in comparison with a constant concentration of the peptide [16]. Bastmeyer *et al.* created ephrinA5 gradients to study the effect of the slope and local concentration of gradients on the growth cone navigation [7]. However, in this study, we did not observe both the alignment and migration of fibroblasts on the hydrogel slice with a concentration gradient of type I collagen, although the attachment and morphology of fibroblasts were strongly influenced by the collagen gradient.

Pelham *et al.* have shown that the cell locomotion and foal adhesions are regulated by the stiffness of PAAm hydrogels [21]. However, unfortunately, we do not evaluate a variation of the substrate stiffness across the hydrogel. In this study, we used PAAm hydrogels with a homogeneous crosslinking, where there is no variation of the substrate stiffness before the treatments. It is possible that the introduction of carboxyl groups in the hydrogel by the hydrolysis reaction may modify both the swelling and the stiffness of the hydrogel by intermolecular repulsion between the carboxyl groups introduced, while this effect could be shielded by the immobilization of type I collagen. Consistent with the shielding effect, we found that the shape of the hydrogel used in the cell culture experiments is not trapezoidal but rectangle (data not shown). In addition, fibroblasts attach well on collagen-immobilized substrates, while poor fibroblasts adhesion was observed for PAAm-grafted substrates as Tamada *et al.* reported [19]. Taken together, we can say with certainly that the attachment and morphology of fibroblasts were strongly influenced by the type I collagen gradient rather than the variation of the substrate stiffness across the hydrogel.

### 2.4. Advantages of the developed technique

Recent advancement of lithographic and micro-manipulation technologies and methodologies allows us to create surface-bound gradient structures with a high resolution, a complex pattern, and a well-designed physicochemical property in two-dimension. The gradient structure can function as a high-throughput screening method to facilitate both the fast screening of physicochemical phenomena and cell behaviors. However, there are some technical limitations on the lithographic and micromanipulation technologies and methodologies to generate three-dimensional gradients even with complex and special equipments. It is therefore clear that an easy and simple technology to form protein gradients in three-dimensional cell scaffolds is greatly required for tissue engineering applications.

Fabrication of three-dimensional protein gradients has emerged in the research field of chemotaxis as an *in vitro* technique to investigate directed cell migration by soluble biomolecules. The Boyden chamber [22] has been employed to study chemotaxis assays, although the gradients generated by the chamber were unstable and hence could not be used for extended time periods. Tissue engineering applications require stabilized multiple gradients of immobilized factors in three-dimensional scaffolds. Immobilized protein gradients can mimic the spatial regulation indispensable for the process of tissue regeneration. To create a three-dimensional gradient of proteins, assembling different microspheres containing proteins with a gradient mixing ratio [14] and mixing different solutions by using a gradient maker normally used to make polyacrylamide gels for electrophoresis [15,16,17], have been investigated. However, there still remain the complex processes in those methods, such as the fabrication of microspheres containing proteins and the synthesis of prepolymers conjugating proteins.

On the contrary, our diffusion-controlled fabrication technique is an easy and simple method based on a side-by-side diffusion chamber, which is applicable to several material types, such as hydrogels, sponges, and non-woven fabrics. We have already succeeded in fabricating sponges and non-woven fabrics with a concentration gradient of both the RGDS peptide and the growth factors, respectively (our unpublished data). In addition, by changing reaction conditions, it is practically possible to alter the profile of protein gradients. A technique similar to our method has been developed to generate immobilization gradients of enzymes in a porous three-dimensional silk fibroin scaffold using the principles of diffusion [23]. Taken together with our data, the diffusion-controlled fabrication technique could be extended to immobilize a variety of proteins and small molecules in several types of porous materials, thereby offering new options in the fields of chemotaxis and tissue engineering.

## 3. Experimental Section

### 3.1. Materials

Acrylamide monomer, *N*,*N*’-methylene-bisacrylamide (BIS), and sodium peroxosulfate, were purchased from Wako Pure Chemical Industries Ltd., Osaka Japan. *N*,*N*,*N*’,*N*’-tetramethylethylenediamine (TEMED), EDC, NHS, and other chemicals were obtained from Nacalai Tesque Inc., Kyoto, Japan. Bovine skin type I collagen solution (3 mg/mL) was supplied from Nitta Gelatin Co., Osaka, Japan. All the chemicals were used without further purification.

### 3.2. Fabrication of hydrogels with a concentration gradient of carboxyl groups

A PAAm hydrogel was prepared by radical copolymerization of acrylamide with BIS. Briefly, an aqueous solution of acrylamide (20 w/v%) containing 6.5 mM BIS and 8.4 mM sodium peroxosulfate was mixed with 100 μL of TEMED, followed by leaving it for 1 h at room temperature to obtain crosslinked PAAm hydrogels. The resulting hydrogel was cut into a disc shape of 8 mm in diameter and 3 mm in thickness and washed with double-distilled water three times.

Hydrogel discs were exposed to a gradient concentration of NaOH solution at 52 °C to generate that of carboxyl groups in the hydrogel disc. The concentration gradient of NaOH was formed in the hydrogel disc by using a side-by-side diffusion chamber (Figure 1), while the hydrogel disc contacted NaOH solution at different concentrations on one side and PBS solution (0.1 M, pH 7.0) on the other side. PBS (1000 mL) circulates through the chamber to achieve an approximate sink condition at pH 7.0. As a control, PAAm hydrogel discs were homogeneously hydrolyzed by a NaOH solution with different pH values at 52 °C for 1 h [24]. The resulting hydrogel disc was washed with PBS three times.

The amount of carboxyl groups generated in the PAAm hydrogel discs was determined by staining with toluidine blue dye according to the method reported previously [24]. Briefly, PAAm hydrogel discs were immersed into an aqueous solution of toluidine blue (2.5 mM, pH 10) for 24 h at room temperature. After extensive washing with NaOH solution (pH 10), the hydrogel disc stained was sliced into sections with a thickness of 25 μm parallel to the surface of the hydrogel disc facing the aqueous solution in the diffusion chamber. Then, 20 slices were collected in order over the hydrogel disc. The dye binding to slices was extracted by an acetic acid solution (50 v/v%) at 100 °C for 2 h. It has been reported that toluidine blue binds to carboxyl groups at a molar ratio of 1.0 [24]. The amount of carboxyl groups generated was calculated by measuring the optical density of the extracts at 633 nm with UV/VIS spectrophotometer (DU-800, Beckman Coulter, Brea, USA).

### 3.3. Fabrication of hydrogel slices with a concentration gradient of type I collagen

The carboxyl groups generated were chemically coupled with the amino groups of type I collagen by using EDC and NHS to prepare hydrogel slices with a concentration gradient of collagen immobilized. Briefly, the PAAm hydrogel disc with a concentration gradient of carboxyl groups was cut longitudinally into a slice (5 mm × 3 mm and 1 mm in thickness). The hydrogel slice was washed with PBS containing 0.14 M sodium chloride, sterilized with 70 vol % ethanol, and washed with a sterile PBS solution containing 0.14 M sodium chloride three times. Then, the carboxyl groups in the hydrogel slice were activated with a sterile PBS solution (pH 7.0) containing EDC (72.0 mM), NHS (35.6 mM), and sodium chloride (0.14 M) for 2 h at room temperature, followed by covalently conjugating with type I collagen under aseptic condition at 4 °C for 12 h. The resulting type I collagen-immobilized hydrogel slice was washed with a sterile solution of hydrochloric acid (1 mM) at 4 °C three times and eventually with Eagle’s minimum essential media (MEM) containing 10 vol % fetal bovine serum three times for subsequent cell culture experiments.

The amount of type I collagen immobilized in the hydrogel slice was determined by staining with SircolTM collagen assay kit (Biocolor Ltd., Newtownabbey, Northern Ireland). Briefly, the hydrogel slice was immersed into the Sircol dye reagent for 30 min at room temperature, followed by extensive washing with double-distilled water. The hydrogel slice stained was viewed on an upright microscope and the intensity of the red signal corresponding to the stained collagen was measured by image analysis of the microscope pictures with ImageJ software (version 1.38, National Institute of Health, USA). Similar to the hydrogel slice, PAAm hydrogels prepared in the presence of type I collagen with known amounts were stained with the Sircol dye reagent to calculate the correlation between the intensity of the red signal and the amount of type I collagen added. The amount of type I collagen immobilized was estimated by comparing the red signal intensity of the hydrogel slice with the calculated correlation.

### 3.4. Evaluation of cell attachment on hydrogel slices with a concentration gradient of type I collagen

The attachment and morphology of mouse fibroblasts (L929) were evaluated for collagen-immobilized hydrogel slice. Briefly, L929 (1 × 105 cells) was seeded onto the hydrogel slice (5 mm × 3 mm and 1 mm in thickness) with a concentration gradient of type I collagen. After 1 day of seeding, the cells attached on the hydrogel slice were viewed on an inverted microscope, and the number of the attached cells was counted. In addition, the cell ellipticity, which was defined as the major to minor axis ratio, was calculated by measuring the length of those axes for the cells attached with ImageJ software [25]. At least 100 cells were subjected to the analysis.

### 3.5. Statistical analysis

All the data were statistically analyzed by using ANOVA, and statistical significance was indicated by a p value less than 0.05. The experimental results were expressed as the mean ± the standard deviation.

## 4. Conclusions

This study indicates that the adhesion behavior of cells can be modified by the concentration gradient of biomolecules in cell scaffolds. The diffusion-controlled fabrication technique is a promising as a method to modify the functional groups of scaffolds with different reactive reagents, which allow scaffolds to chemically modify with biomolecules of concentration gradients.
